# The Language of Genetics In the Interviews of Jane Gitschier

**DOI:** 10.1371/journal.pgen.1006115

**Published:** 2016-06-16

**Authors:** Gregory S. Barsh, Gregory P. Copenhaver

**Affiliations:** 1 HudsonAlpha Institute for Biotechnology, Huntsville, Alabama, United States of America; 2 Department of Genetics, Stanford University School of Medicine, Stanford, California, United States of America; 3 Department of Biology, The University of North Carolina at Chapel Hill, Chapel Hill, North Carolina, United States of America

"Tell me, what is it you plan to do with your one wild and precious life?" The question that concludes Mary Oliver's poem, *The Summer Day*, reminds us not only of a poet inspired by her observations of the natural world but also of Jane Gitschier, a scientist, author, musician, and mother whose relationship with and observations of those around her have contributed so much to our community. This editorial is intended to commemorate and celebrate Jane's series of more than 40 interviews published by *PLOS Genetics*, spanning ten years of publishing, about a billion years of evolution—from Archea to *Brassica*, from prions to mammals—and a set of themes and ideas that make the word "eclectic" seem puny by comparison.

To us, Jane is the Terry Gross of science writing (or, for the younger, more podcast-oriented crowd, Marc Maron), striking a perfect balance in both tone and subject matter. Her conversations are convivial but erudite, illuminating her subject's personality as well as their societal and scientific contributions. Who knew that Mark Ptashne used to listen to Paul Robeson sing at his house [1], that David Botstein used to conduct the Harvard glee club [2], or that Elaine Strass used to be a concert pianist [3]? Reading through Jane's interviews is like being a fly on the wall for a scientific century of genetic ideas and opinions, from the role of lateral gene transfer in evolution (Ford Doolittle [4]) to a "neo-Lamarckian view of biology," which is how Jane describes the impact of Susan Lindquist's work on rapid evolutionary change driven by chaperones and remodeling proteins [5]. Technology also features prominently in Jane's interviews—the nitty-gritty details of the first nitrocellulose blots and the first microarrays are tremendously fun to read in the words of Sir Edwin Southern [6] and Pat Brown [7].

But one of the things we enjoy most about Jane's interviews is that they're not confined to working scientists. Authors (Nicholas Wade [8] and James Schwartz [9]), architects (Rafael Viñoly [10]), CEOs (Anne Wojcicki [11]), and jurists (Judge John E Jones, III [12]) contribute to and, more importantly, expand, the relationship of the genetics community to the rest of the world. Jane explores it all in a way that makes us wish—or perhaps more accurately, feel like—we were there with her during the interviews. In popular culture terms, Jane is someone with whom we would all like to drink a beer (or glass of wine).

Correspondence from Jane often includes Mary Oliver's "wild and precious life" quote in the signature, which may help to explain why Jane's own life has been and continues to be as eclectic as her choice of subjects. Jane's fascination with human genetics and Mendelian disease began during her postdoc at Genentech with Dick Lawn and colleagues and catalyzed her career as a faculty member at the University of California, San Francisco (UCSF) at a time when there was not even a Department of Genetics, let alone Human Genetics (there still isn't, but that's another story). A PubMed search for Gitschier retrieves scientific contributions that read like the table of contents for a human genetics textbook (hemophilia, Menkes' disease, diabetes insipidus, pantothenate kinase–associated neurodegeneration), along with five papers on the genetics of absolute pitch. Indeed, music and musicality feature prominently not only in Jane's interviews and her science but also in her life. Now an emeritus faculty member at UCSF, Jane's current projects include music composition, publishing a book (of her interviews), and going to architecture school. Wild and precious indeed.

Recordings of Jane's interviews have been archived for future use by the American Philosophical Society, which should make for some interesting listening 100 years from now. In addition, all the written interviews will remain as a collection at PLOS, open and freely available to all. In 2010, *PLOS Genetics* published an interview of Jane with herself [13] that concludes with the following:

**Gitschier:** OK, last question. Do you have a favorite interview?
**Gitschier:** …The truth is that each person's story is remarkable in its own way. I can hear all of their voices. I am so grateful to these people who put their trust in me. This project has been one of the most rewarding experiences of my life.

Ours too! Precious, discerning, illuminating, and rewarding.
